# Construction and updating of a public events questionnaire for repeated measures longitudinal studies

**DOI:** 10.3389/fpsyg.2014.00230

**Published:** 2014-03-19

**Authors:** Martha Noone, Maria Semkovska, Mary Carton, Ross Dunne, John-Paul Horgan, Breige O'Kane, Declan M. McLoughlin

**Affiliations:** ^1^Department of Psychiatry and Trinity College Institute of Neuroscience, Trinity College Dublin, St. Patrick's University HospitalDublin, Ireland; ^2^Department of Psychology, University of LimerickLimerick, Ireland

**Keywords:** retrospective memory, semantic memory, episodic memory, general knowledge of the world, public events

## Abstract

Impairments of retrospective memory and cases of retrograde amnesia are often seen in clinical settings. A measure of the proportion of memories retained over a specified time can be useful in clinical situations and public events questionnaires may be valuable in this respect. However, consistency of retention of public events memory has rarely been studied in the same participants. In addition, when used in a research context, public events questionnaires require updating to ensure questions are of equivalent age with respect to when the test is taken. This paper describes an approach to constructing and updating a Public Events Questionnaire (PEQ) for use with a sample that is recruited and followed-up over a long time-period. Internal consistency, parallel-form reliability, test-retest reliability, and secondary validity analyses were examined for three versions of the PEQ that were updated every 6 months. Versions 2 and 3 of the questionnaire were reliable across and within versions and for recall and recognition. Change over time was comparable across each version of the PEQ. These results show that PEQs can be regularly updated in a standardized fashion to allow use throughout studies with long recruitment periods.

## Introduction

Retrograde amnesia refers to loss of previously learned information from before a fixed point or particular occurrence (Lezak et al., 2004). In some cases a fixed point or event cannot be determined, in which case the inability to recall previously learned information is referred to as an impairment of retrospective memory function, instead of retrograde amnesia (Lezak et al., 2004). The study of retrospective memory has generally focused on autobiographical memory and public events memory. Public Events questionnaires (Warrington and Silberstein, 1970; Howes and Katz, 1988; Squire et al., 1989; Reed and Squire, 1998; Meeter et al., 2010) contain questions about memorable events that have occurred over a number of years, some presented firstly in a free recall format, followed by a multiple-choice format to assess recognition memory. Questions may be grouped by time-periods to assess the probable age of the memory according to its recency, for example by grouping memories into 1 or 5 year time-spans and comparing performance on each. Such an approach allows for assessment of both free recall and recognition abilities as well as the effect of passage of time. Public events questionnaires allow for assessment of memories whose details may be independently verified (Warrington and Silberstein, 1970) and are based on common experience across participants (Warrington and Sanders, 1971).

Demographic factors, such as age and gender, have been found to affect performance on public events questionnaires. Age of participant has been examined and it has been found that older adults (65–75) and those under 12 years old perform worse than middle aged participants on these questionnaires (Squire, 1975; Howes and Katz, 1988; Bizzozero et al., 2004). Some authors have noted that while recent memory performance decreased with age, recall of remote memories remained at a constant level across time-periods (Squire, 1975; Howes and Katz, 1988). However, others have shown more remote memories to be more susceptible to forgetting, with the effect more marked for older adults, although present at all ages (Warrington and Sanders, 1971). Gender has been found to affect performance on different categories of question (Howes and Katz, 1988; Meeter et al., 2010), as has interest in media (Meeter et al., 2010).

Consistency of public events memory over repeated assessments has rarely been the focus of studies to date, but two studies that mention this have reported high levels of consistency in healthy controls from one time-point to another (Squire et al., 1989; Bizzozero et al., 2004). However, the issue of consistency was not the primary concern in these studies, and participant numbers were relatively low (*n* = 8–15). A measure of memory consistency is often useful in clinical situations, e.g., elective neurosurgery or radiotherapy, and as such it is important to know how consistent recall is in a normal population.

An important consideration when using these questionnaires is that a new test is generally developed by each research team that uses it (Warrington and Silberstein, 1970; Squire and Slater, 1975; Howes and Katz, 1988; Bizzozero et al., 2004; Meeter et al., 2005) to ensure questions are up to date and tailored for use with the population being assessed. A related issue is the possible need for regular updating of questions because of previous findings that point to a difference in retention rate for older and more recent questions (Warrington and Sanders, 1971; Howes and Katz, 1992). This becomes especially important in longitudinal studies, such as clinical trials, where participants may be recruited over several years and it is necessary to ensure that test items are from equally distant periods of time for all participants and remain balanced in their content. Some authors have looked at methods of updating public events questionnaires (Bizzozero et al., 2004) but to our knowledge only one study has been reported that compares performance between original and updated versions of a public events questionnaire (Meeter et al., 2005). Taken together, these issues highlight the need to develop a method of constructing and easily updating public events questionnaires.

This paper reports a method for constructing a Public Events Questionnaire (PEQ) that can be tailored to specific populations and easily updated every 6 months for use with a sample that is recruited over a long time-period. Reliability analyses of test-retest usage are also reported. Exploratory analyses were carried out to examine validity of the PEQ.

## Materials and methods

### Study sample

Data for this study were collected from 56 healthy participants, 39 of whom were female. Participants completed their first assessments between July 2008 and January 2010 and follow-up assessments between January 2009 and July 2010.

All participants were ≥18 years, had English as first language, and were resident in Ireland, with the majority living in Dublin and surrounding areas. Two participants were excluded from the study because, although resident in Ireland at the time, they had only moved here from abroad in the last couple of years and as such the questions were not culturally relevant.

Participants had Mini Mental State Examination (MMSE) (Folstein et al., 1975) scores of ≥26. Participants had no previous psychiatric history, no cognitive impairment, no current major medical illness or sensory deficit and no alcohol/substance abuse in the previous 6 months. Full informed written consent was obtained prior to commencing the study. Full ethical approval, in accordance with the Declaration of Helsinki, was granted for this study by the local Research Ethics Committee. Volunteers were recruited through advertisements in local businesses, online volunteering websites, social and sports clubs, and Trinity College Dublin.

### Assessment battery

Volunteers completed a cognitive test battery that included measures of estimated IQ [National Adult Reading Test (NART), Nelson and Willison, 1991], global cognitive functioning [Addenbrooke's Cognitive Examination, Revised (ACE-R), Mioshi et al., 2006] incorporating the Mini Mental State Examination (MMSE) and semantic fluency, and the PEQ designed for this study. In addition, most participants completed the Rey-Osterrieth Complex Figure (ROCF, Osterrieth, 1944; Taylor, 1979) as a measure of anterograde visual learning and delayed memory and the Free and Cued Selective Reminding Test (FCSRT, Van der Linden and GREMEM, 2004) as a measure of anterograde verbal learning and delayed memory.

Three versions of the PEQ were used in this study. The versions were updated every 6 months, starting on the first day of January and July each year. Participants were always tested and re-tested on the same version of the questionnaire in order to assess the consistency of performance.

All assessments (except the NART) were administered twice and alternate versions of tests were used where relevant to minimize practice effects.

### The PEQ: formatting

The questionnaire spans the 16 years prior to entering the study. It is split into four time-periods, one pertaining to the most recent 12 months (Events Questionnaire time-period 1- EQ1) and three covering the 15 years before that, divided into three groups of 5-year blocks (EQ5, EQ10, and EQ15). EQ5 contains questions from the 5 year period immediately preceding the most recent 12 months, EQ10 contains questions from the 5 years before that and EQ15 contains questions from the 5 year period before that again.

Each time-period is made up of 15 questions covering three public events categories:

1. National news events
2. International news events
3. Cultural events (including arts, sports, and entertainment)

This results in a total of 60 items for the entire questionnaire. The first version of the questionnaire contained 9 national questions, 23 international questions, and 28 cultural questions. Although other similar questionnaires also contain an uneven number of questions in each category (Warrington and Silberstein, 1970), the PEQ was modified to 20 questions per category overall for Versions 2 and 3 with five questions per category within each time period to ensure an even spread of categories across each time period and enable ease of updating. For EQ 5, 10, and 15 each of the 5 years is assigned three questions, one from each category. This results in a total of 15 questions per time-period. There are also 15 questions in EQ1, all from the same 12 month period, comprising 5 questions from each of the three categories. Questions were sourced from national and international news websites and were randomly distributed across the questionnaire in terms of time-period and category. A pool of questions was sourced from internet news websites for each update of the questionnaire. Prospective questions were discussed at research team meetings and chosen for inclusion in the next questionnaire based on level of memorability (high) and duration of coverage of the event (brief). The research team consisted 3–4 males and 3–4 females at all times, all of whom had a minimum qualification of a third level degree, and were aged between 22 and 49. As such, it is possible that this group were not representative of the participants in the study. However, as consistency with baseline was the main outcome of interest, any differences between those developing the questionnaire and those being tested on the questionnaire should not affect consistency of answering across assessment points. Test items were designed to be clear and succinct and the year of the event in question was included in questions to enhance clarity.

For Versions 2 and 3, EQ1 is made up of two 6 month sections, the *older 6 months* and *most recent 6* months of the 12 month period. When updating, the questions in the *older 6* months of EQ1 (7 or 8 in total) are identified and removed except one question which is chosen arbitrarily to be pushed back into EQ5 in order to update EQ5. It follows that one question must then be pushed out of EQ5 and into EQ10, and out of EQ10 into EQ15, and finally one question will be removed from EQ 15. In order to keep the balance of categories equal across each year, if the question pushed back from EQ1 to EQ5 is a *national* question it follows that all other questions being moved will be *national* questions.

### The PEQ: administration and scoring

Questions were presented orally and participants asked to recall the answer. If unsuccessful at one attempt to recall the answer, they were provided with four possible answers and asked to recognize the correct answer. If at the initial recall stage participants provided a wrong answer that was also one of the incorrect recognition choices, that answer was left out of the recognition choices and they were given a choice of the three remaining answers instead. This was done to avoid repetition of the incorrect answer. Correctly recalled items were scored two points, correctly recognized items scored one point and incorrect items scored zero points. Participants were not told whether they gave the correct answer to minimize a learning effect at 6 month follow-up.

### Data analyses

Differences in demographic factors were assessed between versions. Gender differences were examined using chi-square analysis. NART scores were analyzed using One-Way between group ANOVAs, whereas age and years of education were analyzed using the Kruskal-Wallis non-parametric test as data was not normally distributed. Years of education were computed by adding the average amount of years spent at each completed level of education in Ireland (primary 8 years, secondary 5–6 years, third level 3–4 years, masters 1–2 years, and Ph.D. 3–4 years).

#### Reliability analyses

Internal consistency and parallel-form reliability (based on time period and question category), item difficulty and test-retest reliability (based on time-period only) were assessed. Distribution of question categories differed between Version 1 and Versions 2 and 3. Therefore, for category analyses, raw scores were transformed to percentage correct per category to allow comparisons between Versions. One-Way repeated measures ANOVAs were used to assess internal consistency of each questionnaire by examining differences between categories and between time-periods within each version. Parallel-form reliability was examined by assessing differences between time-periods and categories across versions using One-Way between groups ANOVAs. Bonferroni *post-hoc* comparisons were used.

Item difficulty was examined in two ways and using a more detailed data set where each test item for each participant was coded as to whether the question was recalled, recognized or incorrect/unknown. Firstly, the difference in the amount (count) of answers recalled, recognized or incorrect/unknown across versions was examined using ANOVA. Secondly, the relationship between version and time-period was examined using the Chi-square test for homogeneity of variance (Shaughnessy et al., 2003) to examine whether performance on each time-period was dependent on which version of the PEQ had been administered. The “*n*” referenced here is the total score, that is all answers summed across all versions and all participants, for each time period.

Test-retest reliability was examined using Pearson product moment correlations (*r*) (Pallant, 2007) to investigate correlations between each set of scores (either total score per version or per time period in each version). The amount of change from Time 1 to Time 2 was analyzed by comparing total score at each time point using paired *t*-tests. Differences between change scores (score at Time 2 minus total score at Time 1, Squire et al., 1989) in each time-period in each version were then examined using ANOVA. Significance level was set at *p* < 0.01 to account for multiple testing.

#### Validity analyses

Construct validity of the PEQ was explored by examining correlations (Pearson's product moment correlations—“*r*,” or Spearman's rho for non-parametric data) between the questionnaire and age, IQ and years of education. Convergent and divergent validity was examined through correlations between Public Events Questionnaire results and measures of semantic fluency and delayed visual and verbal memory.

## Results

Three versions of the PEQ were used, starting respectively, on 1st June 2008, 1st January 2009 and 1st July 2009 (Table 1). Results were based on total scores from each time-period (EQ1, EQ5, EQ10, or EQ15), each category (*National, International*, or *Cultural*) or the total score on each version of the PEQ (EQ1+EQ5+EQ10+EQ15). Data are expressed as mean [standard deviation (*SD*)] and were analyzed using SPSS Version 16 (SPSS Inc., 2009).

**Table 1:** **Demographic and education characteristics of study participants**.

**Characteristic**	***n***	**Version 1**	**Version 2**	**Version 3**	**Test statistic**		
					***F*(*df*)**	**χ2 (*df*)**	***p***
		*n* = 25	*n* = 14	*n* = 17			
Female *n (%)*	56	20 (80)	13 (93)	6 (35)		14.32 (2)	0.001
NART	56	111.36 (6.1)	111.79 (4.7)	117.41 (4.6)	6.27 (2)		0.004
Age (years)	56	34.44 (11.9)	48.75 (13.3)	39.53 (12.5)	13.06 (2)		0.001
Education (years)	50	16.76 (1.9)	17.5 (0.8)	17.0 (1.6)	0.953 (2)		0.621

*Data are expressed as mean (sd)*.

Gender, age, and NART scores were significantly different across versions (Table 1). NART scores on Version 3 were significantly higher than those on both Versions 1 and 2, which did not differ significantly. No differences were found between Versions for years of education.

### Reliability

#### Internal consistency

On Version 1 of the questionnaire scores on EQ10 were significantly higher than those in EQ 1 (*p* = 0.004), EQ 5 (*p* = 0.001), and EQ 15 (*p* = 0.002); all others were comparable (see Table 2A). No significant differences were found between time-periods within either Version 2 or Version 3.

**Table 2:**
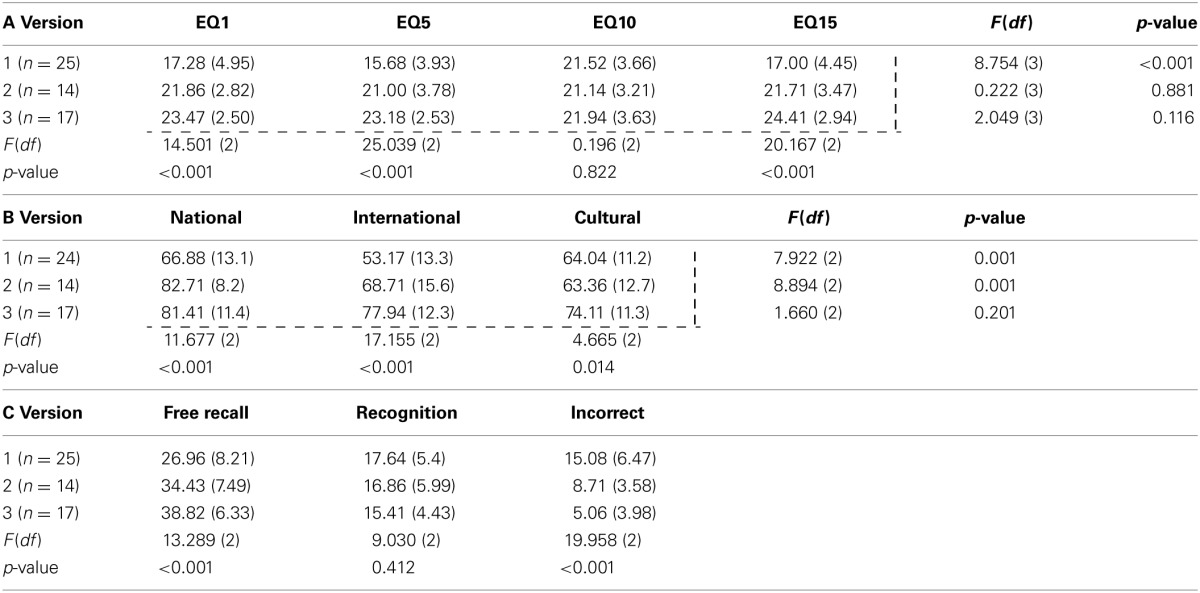
**PEQ scores on Time-period and Category**.

*(A) Parallel form (↓) and internal consistency (→) reliability by time-period. (B) Parallel form (↓) and internal consistency (→) reliability by category (% correct). (C) Parallel form (↓) reliability by answer type (recall/recognition/incorrect or unknown). All data shown as mean (sd)*.

On Version 1 scores for *International* questions were significantly lower than *National* questions (*p* = 0.001), while the difference approached significance between *International* and *Cultural* questions (*p* = 0.012). *National* and *Cultural* questions did not significantly differ. A significant difference on categories was also found for Version 2 where scores for *National* questions were significantly higher than *Cultural* questions (*p* = 0.001), while the difference approached significance between *National* and *International* questions (*p* = 0.016). No differences were found between categories for Version 3 (see Table 2B).

#### Parallel-form reliability

A significant difference in scores was shown across versions for EQ1, EQ5, and EQ15 (Table 2A). Bonferroni *post-hoc* analyses indicated that scores on Version 1 were significantly lower than those on Version 2 for EQ1 (*p* = 0.002), EQ5 (*p* ≤ 0.001), and EQ15 (*p* = 0.002) as well as on Version 3 EQ1 (*p* ≤ 0.001), EQ5 (*p* ≤ 0.001), and EQ15 (*p* ≤ 0.001); however, Versions 2 and 3 were comparable on all time periods.

Performance on *National* questions was significantly different across versions with scores on Version 1 significantly lower than Versions 2 (*p* ≤ 0.001) and 3 (*p* = 0.001) while no difference was detected between Versions 2 and 3 using Bonferroni *post-hoc* testing. A significant difference in performance on *International* questions was also found whereby scores in Version 1 were found to be significantly lower than Versions 2 (*p* = 0.004) and 3 (*p* ≤ 0.001), with no difference detected between Versions 2 and 3 in *post-hoc* analyses. Performance on the *Cultural* category was not significantly different across versions, but approached it at *p* = 0.014.

#### Item difficulty: recall, recognition, and incorrect answering

Significant differences were found between total amount of answers recalled between Versions 1 and 2, and also between Versions 1 and 3, with a significantly lower amount recalled in Version 1 (see Table 2C). There was no significant difference in the amount of answers recognized across versions. The number of questions answered incorrectly, or not known, was significantly different across versions, with a higher amount incorrectly answered on Version 1 than either Versions 2 or 3, which did not differ significantly.

#### Item difficulty: time-period

A chi-square test for homogeneity was used to examine whether PEQ Version and answer type were related, indicated by Cramer's V and associated significance level (*p*). Version and answer type were shown to be significantly related for EQ1: χ^2^_(4, *n* = 837)_ = 0.178, *p* ≤ 0.001; EQ5: χ^2^_(4, *n* = 828)_ = 0.221, *p* ≤ 0.001; and EQ15: χ^2^_(4, *n* = 839)_ = 0.225, *p* ≤ 0.001, while EQ10 approached significance at EQ10: χ^2^_(4, *n* = 836)_ = 0.082, *p* ≤ 0.024.

#### Test-retest reliability and consistency over time

Pearson product-moment correlations between baseline and 6 month follow-up scores were carried out for total score and then for each time-period on each version (Table 3). Positive correlations were found between baseline and 6 month follow-up total scores, exceeding *r* = 0.80, *p* < 0.001, for all versions which is a desirable correlation in test-retest reliability (Pallant, 2007). All time-period correlations were above 0.70 and significant at *p* < 0.01, except for EQ1, Version 2 which was significant at *p* < 0.05 (*p* = 0.011).

**Table 3:** **Pearson product-moment correlations (*r*) between baseline and 6 month follow-up**.

**Version**	**Total score**	**EQ1**	**EQ5**	**EQ10**	**EQ15**
	*r*	*r*	*r*	*r*	*r*
1 (*n* = 18)	0.959	0.916	0.905	0.823	0.926
95% C.I.	0.892–0.984	0.785–0.968	0.759–0.964	0.579–0.931	0.809–0.972
2 (*n* = 13)	0.906	0.678	0.831	0.812	0.733
95% C.I.	0.709–0.971	0.203–0.894	0.517–0.947	0.473–0.941	0.306–0.914
3 (*n* = 15)	0.899	0.700	0.767	0.725	0.805
95% C.I.	0.717–0.916	0.293–0.892	0.420–0.918	0.339–0.902	0.499–0.932

**Figure 1:**
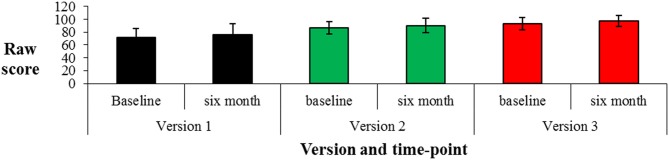
**Scores on each version [mean (*sd*)] at baseline and 6 month follow-up on each version of the Public Events Questionnaire**.

The *amount* of change from baseline to 6 month follow-up was examined using paired *t*-tests (Figure 1). A significant difference was found between baseline and 6 month follow-up performance for Version 1 [*t*_(17)_ = −3.721, *p* = 0.002], Version 2 [*t*_(12)_ = −2.867, *p* = 0.014] and Version 3 [*t*_(15)_ = −4.720, *p* < 0.001] with an average increase of between 3.7 and 4.4 points at follow-up (Figure 1). The *difference* between the change scores for each time-period across versions was then investigated and was not significant [EQ1 *F*_(2, 43)_ = 0.226, *p* = 0.799; EQ5 *F*_(2, 43)_ = 0.053, *p* = 0.948; EQ10 *F*_(2, 43)_ = 0.505, *p* = 0.607; EQ15 *F*_(2, 43)_ = 0.474, *p* = 0.626], again indicating change over time was comparable for each time-period across versions.

### Validity

#### Demographic indicators

A significant correlation was found between performance on the Events Questionnaire and NART scores for Version 1 total score (*n* = 25, *r* = 0.639, *p* = 0.001), EQ1 (*n* = 25, *r* = 0.582, *r* = 0.002), and EQ15 (*n* = 25, *r* = 0.625, *p* = 0.001) but no other significant correlations were found between individual versions and age, education or other NART scores.

**Figure 2:**
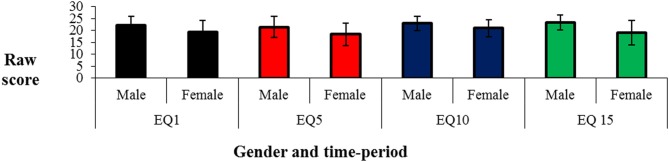
**Scores [mean (*sd*)] on the Public Events Questionnaire by gender for time-period**.

**Figure 3:**
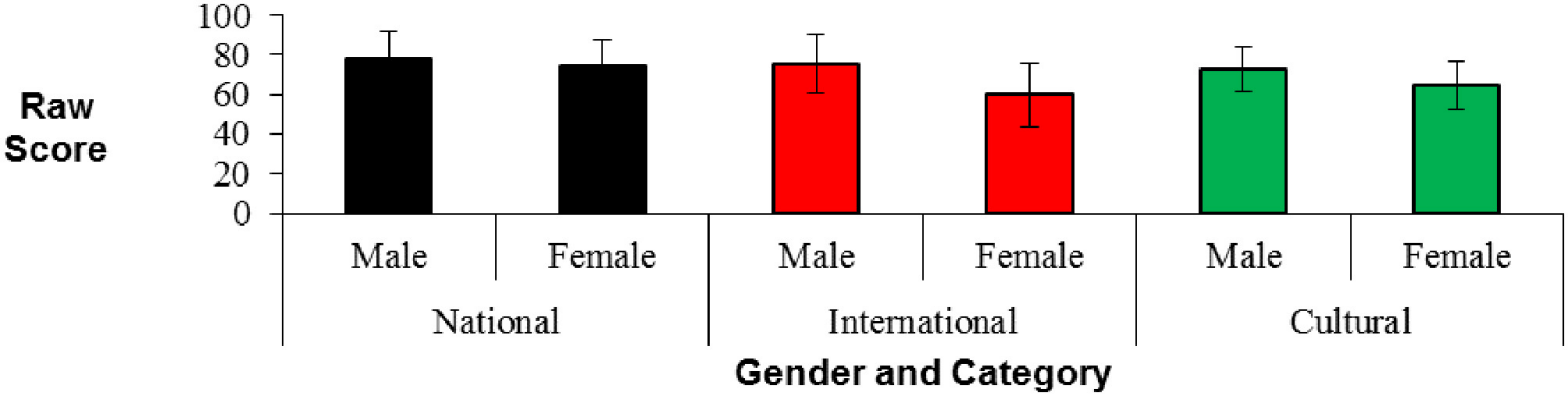
**Scores [mean (*sd*)] on the Public Events Questionnaire by gender for category**.

Versions 1 and 3 only were examined for gender differences, as there was only one male in the Version 2 group. No significant differences were found between performances of males and females on any time-period or category for Version 1 or Version 3 (see Figures 2, 3).

#### Convergent/divergent validity

The relationship between public events and semantic memory was also examined. Version 2 was not included in these correlations as there were only 8 participants in that group. No significant correlations were found between semantic fluency and total score on Version 1 (*n* = 25, *r* = 0.250, *p* = 0.228) or Version 3 (*n* = 17, *r* = 0.083, *p* = 0.753). Correlations between the PEQ and delayed verbal and visual memory, as measured by the FCSRT delayed recall task and the ROCF delayed recall, respectively, were examined for Version 1 only, as participants in Version 3 did not complete all tests. This was a result of shortening of the test battery in order to recruit as many controls as possible. Due to the length of the study, recruitment was proving more difficult for the third version. No significant correlation was found between Version 1 and delayed verbal recall (*n* = 25, *r* = 0.118, *p* = 0.574) and no significant correlation was found between delayed visual recall and scores on Version 1 (*n* = 25, *r* = 0.012, *p* = 0.953).

The relationship between free recall on the PEQ (a score of “2”) and free delayed recall on the FCSRT was carried out for Version 1 (Versions 2 and 3 had *n* = 8). No significant correlations were found for either total score or each time-period on Version 1. The relationship between total cued recall on the FCSRT and cued recognition on the PEQ (a score of “1”) was also investigated and again no significant relationship was shown either for total score or each time-period in Version 1.

## Discussion

We present here an approach toward generating a Public Events Questionnaire for use in longitudinal studies to assess consistency of public events memory while also allowing for the PEQ to be updated in a standardized fashion. The three versions of the PEQ examined here showed similar consistency of memory recall and recognition when retested after 6 months. However, despite reasonable reliability findings there were some limitations regarding validity.

In order to understand changes in clinical populations the efficacy of the PEQ in examining consistency in public events memory over time needed to be assessed in a healthy control population first. Analyses of test-retest reliability with the current healthy participants demonstrated consistent performance from baseline to follow-up assessment for all three versions of the PEQ. Although there was an increase in scores from initial assessment to follow-up on each version of the questionnaire this did not differ across versions and suggest a stable practice effect upon repeated testing. These findings are in line with those from two earlier studies that found strong correlations for test-retest reliability in public events memory (Squire, 1975; Bizzozero et al., 2004) and indicate that this PEQ is a reliable method of assessing consistency of public events memory over time. This knowledge should prove useful when making clinical decisions about what is normal or not in terms of memory change over time.

In terms of comparing different time-periods within the questionnaire, Versions 2 and 3 were found to be reliable across time-periods and Version 1 showed performance in three of four time-periods to be reliable. The change after Version 1 to a more standardized method of test construction in Versions 2 and 3 resulted in improved reliability here. Reliability within each version allows for comparison of a participants performance across each time-period, on questions of differing age. This may prove useful when examining whether recent or more remote memories have been affected in clinical populations.

Performance on time-periods across versions was comparable for Versions 2 and 3; however, performance on three out of four time-periods on Version 1 was significantly worse than on equivalent time-periods in other versions. Again, this points to improved parallel-form reliability in Versions 2 and 3 using the standardized method outlined. However, assessing reliability by categories led to more varied results. When the mean percentage correct is examined across studies the raw data a trend can be seen whereby in all three questionnaires participants performed best on National questions (Table 2B) and, for Versions 2 and 3, the second best performance was in International questions and the worst performance on Cultural questions.

Item difficulty was analyzed and findings showed that Version 1 had fewer recalled answers and more incorrect answers than Version 2 or 3. Taken together with findings outlined above, this suggests that Version 1 may have been more difficult than later versions. However, question difficulty may not have been the only reason for this difference. In previous studies higher IQ has been shown to be associated with better performance on events questionnaires (Squire, 1975; Howes and Katz, 1988). In the current study significant correlations were found between NART scores and PEQ performance on Version 1 for total score and for the oldest and most recent time-periods, pointing to an influence of IQ upon those time-periods that have been shown to be most susceptible to forgetting (Warrington and Sanders, 1971; Squire and Slater, 1975; Howes and Katz, 1988). However, this relationship was not found in Version 2 or 3 and therefore it is difficult to say how much of an effect IQ had on performance here.

Validity analyses were limited due to small numbers of participants in Versions 2 and 3 who completed all assessments. It was also limited due to the lack of a comparison public events questionnaire to show convergent validity. However, as noted above, this would have been difficult to do as many research teams must develop their own questionnaire. Nevertheless, a measure of general knowledge memory, such as the information subtest on the Wechsler Adult Intelligence Scale, 4th edition (Wechsler, 2009), could have been useful in this respect. In place of this, measures of delayed visual and verbal memory were used as markers of long-term memory in general. In addition, no autobiographical memory questionnaire was used here, which could possibly have shown divergent validity from public events memory. Semantic fluency was measured but no other tests of executive functioning were used, again limiting findings in this section. The only relationship found with the PEQ was with the NART on Version 1, as discussed above. Taken together, these validity analyses were limited and therefore do not allow for conclusions to be drawn as to the type of memory processes which may or may not be involved in recall of public events memories at this time.

This study has some limitations such as the small sample sizes and the differences between age, gender, and NART scores across versions. With regard to small sample size a power calculation was not originally carried out as this was a pilot study of the new questionnaire model. Socioeconomic status was not reported in the current study, however, NART performance and level of education were reported instead. Another limiting factor is the change in format between Version 1 and Versions 2 and 3. However, this resulted in improved reliability on time-periods and categories across versions and suggests that following the standardized procedure for updating the questionnaire leads to better reliability. It may also be noted that in terms of test item construction the year of the event in question was included in questions which may have aided participants somewhat. However, as the goal was to look at consistency of recall it was felt better to have specific questions that removed ambiguity and enhanced clarity at all times. Lastly, there is the issue of practice effects when re-testing. The nature of the question of interest in this study (i.e., does the participant still remember what they remembered at initial testing?) makes it impossible to use an alternate version of the questionnaire at subsequent assessments. However, this effect was stable across each version of the PEQ and thus can be taken into account when re-testing.

Consistency of public events memory over repeated assessments has rarely been studied despite the use of such questionnaires since the 1970's for assessment of long-term memory. A measure of memory consistency is often useful in clinical situations, such as before and after elective neurosurgery or radiotherapy, and when monitoring changes to memory as may be useful in memory clinics and electroconvulsive therapy settings. As such it is important to know how consistent recall is in a normal population in order to interpret change in a clinical population and the results of this study suggest that, despite a remarkably similar practice effect for each version, recall and recognition of public events memories remain quite stable over a period of 6 months.

When using such questionnaires in longitudinal studies, such as clinical trials, it is necessary to ensure that test items are regularly updated and balanced in their content. Future studies that aim to assess verifiable long-term memory consistency would be encouraged to develop public events questionnaires in a similar manner to the method outlined here to ensure parallel forms of test materials are reliable.

## Author contributions

The PEQ was developed and designed by Ross Dunne, Maria Semkovska, Martha Noone and Declan M. McLoughlin. Participants were recruited and assessed by Martha Noone, Mary Carton, John-Paul Horgan and Breige O'Kane. Statistical analysis was guided by Maria Semkovska and carried out by Martha Noone. The main body of text was written by Martha Noone with Maria Semkovska and Declan M. McLoughlin contributing to important revisions.

### Conflict of interest statement

The authors declare that the research was conducted in the absence of any commercial or financial relationships that could be construed as a potential conflict of interest.

## References

[B1] Bizzozero I. Lucchelli F. Prigione A. Saetti M. C. Spinnler H. (2004). What do you remember about Chernobyl? - A new test of memory for media-mediated events. Neurol. Sci. 25, 205–215 10.1007/s10072-004-0323-3 15549506

[B2] Folstein M. F. Folstein S. E. McHugh P. R. (1975). Mini-mental state: a practical method for grading the cognitive state of patients for the clinician. J. Psychiatry Res. 12, 189–198 10.1016/0022-3956(75)90026-6 1202204

[B3] Howes J. L. Katz A. N. (1988). Assessing remote memory with an improved public events questionnaire. Psychol. Aging 3, 142–150 10.1037/0882-7974.3.2.142 3268252

[B4] Howes J. L. Katz A. N. (1992). Remote memory: recalling autobiographical and public events from across the lifespan. Can. J. Psychol. 46, 92–116 10.1037/h0084311 1591652

[B5] Lezak M. D. Howieson D. B. Loring D. W. (2004). Neuropsychological Assessment. New York, NY: Oxford University Press

[B6] Meeter M. Murre J. M. J. Janssen S. M. J. (2005). Remembering the news: modeling retention data from a study with 14,000 participants. Mem. Cogn. 35, 793–810 10.3758/BF03193075 16383168

[B7] Meeter M. Ochtman D. J. C. Janssen S. M. J. Murre J. M. J. (2010). Of sports and politics: predicting category-specific retention of news events from demographic variables. Eur. J. Cogn. Psychol. 22, 117–129 10.1080/09541440802708037

[B8] Mioshi E. Dawson K. Mitchell J. Arnold R. Hodges J. R. (2006). The Addenbrooke's Cognitive Examination Revised (ACE-R): a brief cognitive test battery for dementia screening. Int. J. Geriatr. Psychiatry 21, 1078–1085 10.1002/gps.1610 16977673

[B9] Nelson H. E. Willison J. R. (1991). The Revised National Adult Reading Test- Test Manual. Windsor: NFER-Nelson

[B10] Osterrieth P. A. (1944). Le test de copie d'une figure complexe. Arch. Psychol. 30, 206–356 17007938

[B11] Pallant J. (2007). SPSS Survival Manual. Maidenhead: Open University Press

[B12] Reed J. M. Squire L. R. (1998). Retrograde amnesia for facts and events: findings from four new cases. J. Neurosci. 18, 3943–3954 9570821 10.1523/JNEUROSCI.18-10-03943.1998 PMC6793126

[B13] Shaughnessy J. J. Zechmeister E. B. Zechmeister J. S. (2003). Research Methods in Psychology. New York, NY: McGraw-Hill

[B14] Squire L. R. (1975). A stable impairment in remote memory following electroconvulsive therapy. Neuropsychologia 13, 51–58 10.1016/0028-3932(75)90047-0 1109461

[B15] Squire L. R. Haist F. Shimamura A. P. (1989). The neurology of memory - quantitative assessment of retrograde-amnesia in two groups of amnesic patients. J. Neurosci. 9, 828–839 2926483 10.1523/JNEUROSCI.09-03-00828.1989 PMC6569980

[B16] Squire L. R. Slater P. C. (1975). Forgetting in very long-term memory as assessed by an improved questionnaire technique. J. Exp. Psychol. Hum. Learn. Mem. 1, 50–54 10.1037/0278-7393.1.1.50

[B17] Taylor L. B. (1979). Psychological assessment of neurosurgical patients, in Functional Neurosurgery, eds Ramussen T. Marino R. (New York, NY: Raven Press), 165–180

[B18] Van der Linden M. GREMEM (2004). L'évaluation Des Troubles De La Mémoire- Présentation De Quatre Tests De Mémoire Épisodique (Avec Leur Étalonnage) [Memory Disorders Assessment-Four Episodic Memory Tests With Normative Data]. Marseille: Solal

[B19] Warrington E. K. Sanders H. I. (1971). The fate of old memories. Quart. J. Exp. Psychol. 23, 432–442 10.1080/14640747108400255

[B20] Warrington E. K. Silberstein M. (1970). A questionnaire technique for investigating very long term memory. Q. J. Exp. Psychol. 22, 508–512 10.1080/14640747008401927

[B21] Wechsler D. (2009). Wechsler Memory Scale. 4th Edn San Antonio, TX: Pearson

